# Breakage of transgenic tobacco roots for monoclonal antibody release in an ultra-scale down shearing device

**DOI:** 10.1002/bit.25006

**Published:** 2013-09-20

**Authors:** Sally Hassan, Eli Keshavarz-Moore, Julian Ma, Colin Thomas

**Affiliations:** 1 Department of Biochemical Engineering, University College LondonLondon, United Kingdom; 2 Molecular Immunology Unit, Division of Clinical Sciences, St. George's University of LondonLondon, United Kingdom; 3 School of Chemical Engineering, University of BirminghamWest Midlands, United Kingdom

**Keywords:** transgenic tobacco, roots, shear device, antibody

## Abstract

Transgenic tobacco roots offer a potential alternative to leaves for monoclonal antibody (MAb) production. A possible method for extraction of MAbs from roots is by homogenization, breaking the roots into fragments to release the antibody. This process was assessed by shearing 10 mm root sections (“roots”) in a 24 mL ultra-scale down shearing device, including an impeller with serrated blade edges, intended to mimic the action of a large-scale homogenizer. Size distributions of the remaining intact roots and root fragments were obtained as a function of shearing time. The data suggest that about 36% of the roots could not be broken under the prevailing conditions and, beyond these unbreakable roots, the fragmentation was approximately first order with respect to intact root number. It was postulated that root breakage in such a high shearing device was due to root-impeller collisions and the particle size data suggest that roots colliding with the impeller were completely fragmented into debris particles of the order of 0.1 mm in length. IgG release normalized to release by grinding appeared to lag behind the number of roots that had fragmented, suggesting that a process of leakage followed fragmentation in the ultra-scale down shearing device.

Although mammalian cell culture remains the main expression system for monoclonal antibodies, transgenic plants offer a possible alternative (Ahmad et al., 2012). Complex full-length immunoglobulins have been successfully expressed in transgenic tobacco (Ma et al., 1994) and offer advantages such as low upstream costs and low risk of mammalian virus contamination (Drake et al., 2003; Sharma and Sharma, 2009). Examples of tobacco plant-derived pharmaceutical products currently in clinical development produced using approved GMP-complaint processes include the antibody P2G12 with indications as a HIV microbicide, developed by the Pharma-Planta Consortium, now in phase I clinical trials (Fischer et al., 2012) and scFv antibody fragments for the treatment of non-Hodgkin Lymphoma expressed in tobacco leaves infected with tobacco mosaic virus vectors, produced by Kentucky Bioprocessing, LLC, with phase I clinical trials completed (McCormick et al., 2008). Here, the extraction (pre-purification) from transgenic tobacco of Guy's 13 MAb was studied. This acts against *Streptococcus mutans* (van Dolleweerd et al., 2003), the main causative agent of tooth decay in the mouth. Most of the literature describing monoclonal antibody (MAb) production from *Nicotiana tabacum* plants has involved its extraction from fresh leaf tissue (Platis et al., 2008; Ma et al., 2003), largely because tobacco leaves represent the majority of the total plant biomass. However, the extraction of the MAb from tobacco roots may also be a viable alternative, since roots show similar IgG levels to the leaves per unit fresh mass (Hassan et al., 2008a), and also contain lower levels of toxic phenolics and alkaloids. The nicotine level in tobacco leaves, for example, is three times that in the roots (Dawson and Solt, 1959), thus potentially posing a greater burden on downstream processing.

To date the physical breakage of transgenic tobacco roots has not been considered as a potential system for MAb production although it was suggested by Hassan et al. (2008a). Grinding in liquid nitrogen, denoted by Hassan et al. (2008b) as the “gold standard” for maximal IgG release from transgenic tobacco leaves at bench-top scale and used here as a control for release from roots, is not suitable for large scale operations. The alternative of using a shearing device to release IgG from tobacco roots has been investigated here using a custom built device based on established equipment (Boychyn et al., 2001) modified by the use of an impeller with serrated edges. The intention was to mimic the action of a large-scale homogenizer, with the assumption that this is a scalable device due to both its geometry and operating conditions. This device also had similarities to the scalable mixer device, 088/150 UHS Silverson rotor-stator reported by Hall et al. (2011). Since a large amount of transgenic tobacco roots was not available, it was decided that this was a useful tool to investigate how an IgG1 MAb might be released from the roots of transgenic tobacco plants.

Ten millimeters root sections (“roots”) were sheared in the device. Table I shows the number of intact roots remaining after various shearing times, and IgG release normalized to grinding in liquid nitrogen. In theory, the initial mean fraction of intact roots should be 1 but these roots were treated exactly the same as at other shearing times, and following centrifugation and re-measurement the mass of intact roots was slightly less than the initial mass. The fraction of remaining intact roots decreased with shearing time up to 120 s, after which there was no significant change. The fragmentation was very rapid and, ideally, shearing times less than 30 s would have been investigated in more detail. However, it took several seconds for the device to reach full speed and such data is likely to have been unreliable. The results show that a significant fraction of the roots were not very susceptible to damage at the prevailing conditions. Equation 1 described in Materials and Methods Section, was fitted to the data with SPSS (IBM) using the fraction of unbreakable roots *u* and a breakage constant *ηf* as adjustable parameters, and the fitted values were 0.36 ± 0.06 and 0.032 ± 0.016 s^−1^ respectively. (Unless otherwise stated, errors quoted in this paper are standard error of the mean, SEM.) It appears that the model fits the data well, even though the coefficient of variation for the breakage constant is high.

**Table I:** Mean fraction of remaining intact roots and IgG release after shearing for times up to 360 s at 75 s^−1^ in the shearing device

Shearing time (s)	Mean fraction of intact roots after shearing	Model prediction of fraction of intact roots	IgG release (fraction of nitrogen grinding)	Model prediction of fractional IgG release
0	0.92 ± 0.05	1.00	0.1 ± 0.1	0.2
30	0.60 ± 0.07	0.61	0.3 ± 0.2	0.8
120	0.38 ± 0.03	0.37	0.8 ± 0.4	1.2
240	0.35 ± 0.06	0.36	2.3 ± 0.8	1.3
360	0.36 ± 0.04	0.36	1.0 ± 0.8	1.3

IgG release was expressed as a fraction of IgG release by grinding in liquid nitrogen from roots of the same plant. The experimental measurements were done in triplicate and the estimate of the errors in each case was ± the standard error of the mean (SEM). Corresponding model predictions from Equation 1 of the fraction of intact roots after shearing are also given. The fraction of unbreakable roots, *u*, was found by fitting the curve, to be 0.36 and the breakage constant, *ηf*, was 0.032 s^−1^. Model predictions are also given for IgG release, assuming the latter was proportional to the number of fragmented roots. The proportionality constant was found by fitting the curve, to be 2.

Hassan et al. (2008a) demonstrated by micromanipulation that lignin was the main cause of an increased breakage force of older root types (the 38% of roots that were light or dark brown). The mean breaking force of these roots was 148 ± 29 mN compared to that of the lighter colored roots of 89 ± 14 mN. However, the wide distributions of breaking force meant that some white roots were stronger than some darker ones. Nevertheless, it is speculated that the unbreakable roots in this study were those containing more lignin. Separation by root color was not attempted in the present experiments as that would not be a sensible approach for large scale MAb extraction. Although further investigation is needed to discover why some roots appeared to be unbreakable under the test conditions, IgG release (Table I) suggests that after 120 s, all or nearly all the IgG had been released, indicating that the roots were sufficiently sheared for the purposes of this investigation. In general IgG release increased with time (Table I), presumably because it depends on root fragmentation. The standard error of the mean of these data is large because the IgG measurements both after shearing and after grinding were very variable. Hassan et al. (2008a) indicated that IgG levels in transgenic tobacco roots had a high degree of variability, with a similar finding for leaves by Hassan et al. (2012). It is likely that variability shown in Table I was inherent in the plants rather than just caused by experimental errors.

Assuming that shearing cannot release more IgG than grinding, it can be hypothesized that IgG release was proportional to the number of roots that had been fragmented. The proportionality constant was found to be about 2 by fitting in Microsoft Excel. Although, no strong conclusion can be drawn regarding IgG release because of the variability, there are indications in Table I that IgG release may have lagged root fragmentation to some degree, which would be consistent with roots cells being broken and then subsequent leakage of the antibody from them.

Interestingly, the distributions of lengths of roots and fragments after all shearing times were bimodal and were dominated in number by the fragments. Figure 1a shows a combined frequency distribution of lengths of roots and fragments after 120 s of shearing, as an example. The distributions strongly suggest breakage of roots was by complete fragmentation of previously intact roots, presumably on collision with the impeller. Figure 1b is an expansion of Figure 1a, for fragments less than or equal to 0.2 mm. The mean fragment length (i.e., excluding intact roots only) for the combined data at all shearing times was 0.135 ± 0.003 mm and the mode was 0.064 mm. It can be inferred that each root broke into 74 fragments on average. By microscopic examination of the roots, the size of a typical cell was determined to be about 100 µm × 40 µm. This and the data in Figure 1 suggest fragmentation was into single cells or small cell aggregates, with accompanying debris.

**Figure 1:**
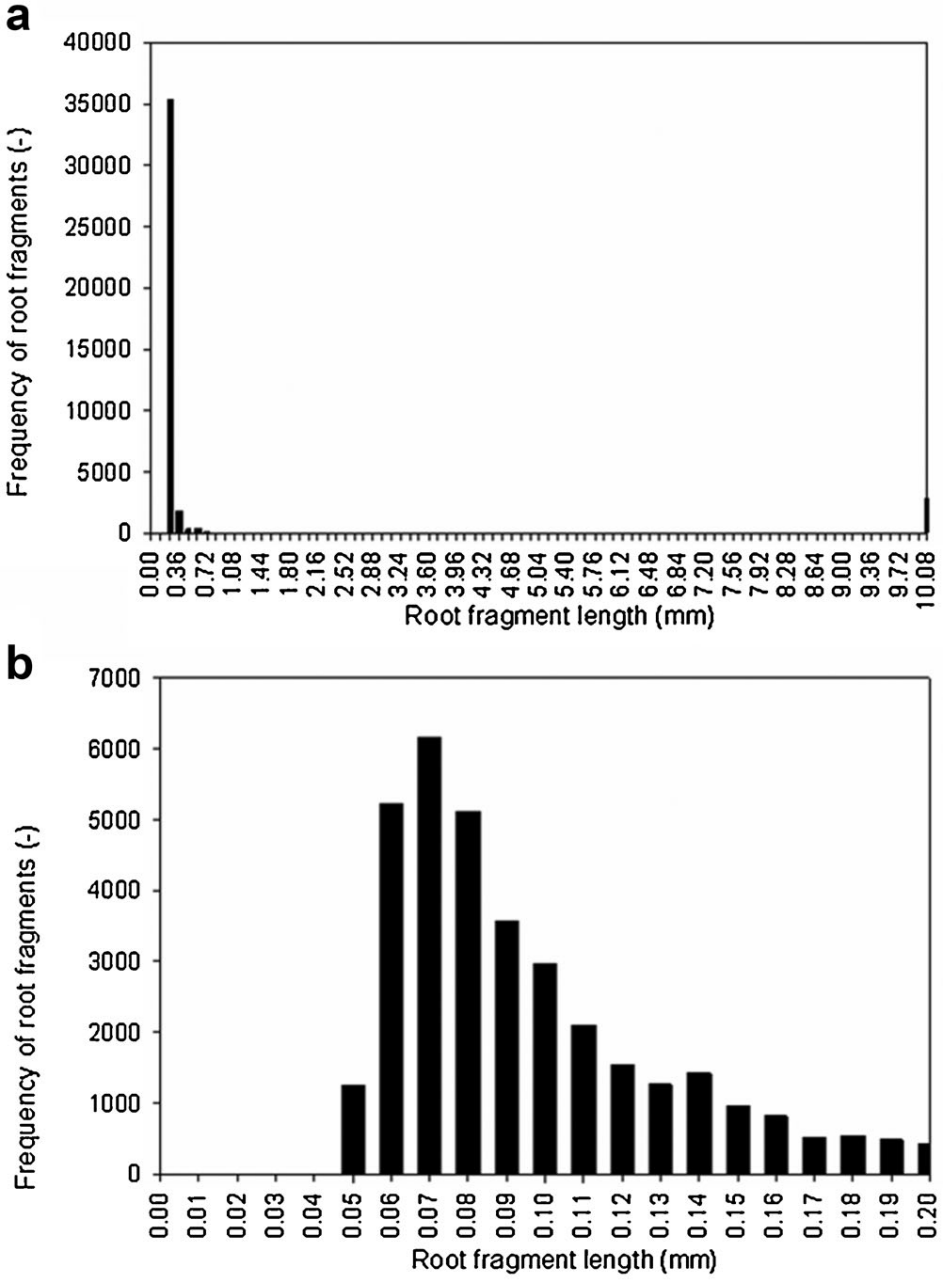
Histogram of lengths of (a) roots and fragments after 120 s shearing at 75 s^−1^ in the shearing device, showing a typical bimodal distribution, (b) fragment lengths for fragments up to 0.2 mm in length.

It was useful to calculate the combined collision and breakage factor *η* from the breakage constant *ηf* already determined to be 0.032 ± 0.16 s^−1^. The impeller Power number used in Equation 4 to estimate the frequency of passage of suspension through the impeller, *f* was determined to be approximately 0.11. This is comparable to that for the EkatoMizer®, a saw-tooth impeller, with an impeller diameter of 0.095 m, a thickness of 0.003 m, and two blades of thickness 0.025 m, where the estimated power number was similarly very low, that is, 0.095 (Xie et al., 2007). Assuming *Po* = 0.11, *D* = 3 × 10^−2^ m, *W* = 1.5 × 10^−3^ m, *N* = 75 s^−1^, *V* = 7 mL = 7 × 10^−6^ m^3^, then *f* ∼ 5 s^−1^. This is not a surprising value given the large impeller to tank diameter ratio. The combined collision and breakage factor *η* was about 0.006, that is, 1 in about every 160 passages of a root through the impeller resulted in breakage. This seems reasonable for nearly neutrally buoyant particles that would be expected to follow the flow closely, despite their lengths.

## Materials and Methods

### Plant Material Preparation

Seven transgenic plants expressing Guy's 13 MAb (secreted IgG1) and two wild type *N. tabacum (var. xanthii)* plants were grown in soil from seed. The exact preparation and screening of transgenic plants are described elsewhere (Drake et al., 2003). All plants used in experiments were young, that is, at pre-flowering stage, and had a mean height of 23 ± 6 cm. Roots rinsed with water were cut into 10 mm sections and suspended in phosphate buffered saline (1× PBS) at a concentration of ca. 5% (w/v), that is, about 0.35 g of root pieces in 7 mL of PBS. The exact weight was recorded in each case. Since a single plant did not possess enough roots for a single shearing experiment, roots taken from several plants were used to make up the 0.35 g required for a single shearing time.

### Design of the Shearing Device

**Figure 2:**
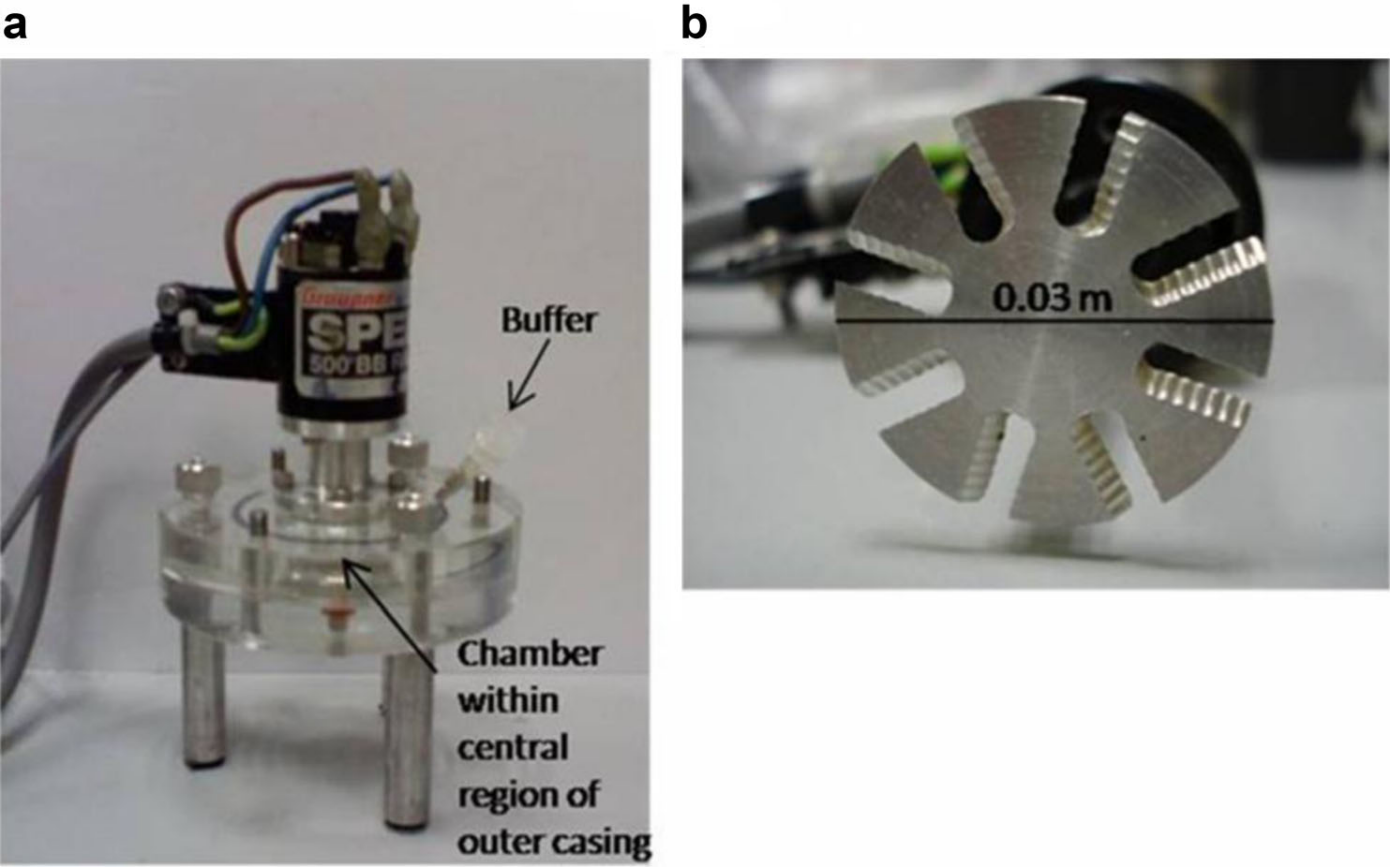
a: The custom built shearing device used to extract IgG from transgenic tobacco roots. b: The impeller consists of eight blades each with two serrated edges.

The shearing device was a flat-bottomed, cylindrical mixing tank made from Perspex (Fig. 2); the actual outer casing on this device was very large, so that the internal diameter and the total height of the chamber were 0.055 and 0.01 m respectively. The impeller diameter was 0.03 m giving a large impeller to tank diameter ratio (0.545) which meant that high radial flow velocities could be achieved. The impeller consisted of eight blades with a serrated edge with a blade width of 0.008 m and a thickness of 0.0015 m.The chamber volume and suspension volume were 24 and 7 mL, respectively. An impeller rotational speed of 75 s^−1^ was chosen since it was above the visually determined minimum speed for root suspension, that is, the minimum impeller speed required to keep the roots suspended in the vessel for longer than 1–2 s (Zwietering, 1958). The Reynolds number at this speed was 6.8 × 10^7^, implying turbulent flow. In order to estimate the (mean) frequency of passage of the suspension through the impeller, *f*, it was necessary to determine the Power number for the novel impeller geometry used in these experiments. Hence this was measured as described in Gill et al. (2008). The measurements were carried out in triplicate, at two different speeds.

### Root Breakage Experiments and the Estimation of the Percentage of Intact Roots

**Figure 3:**
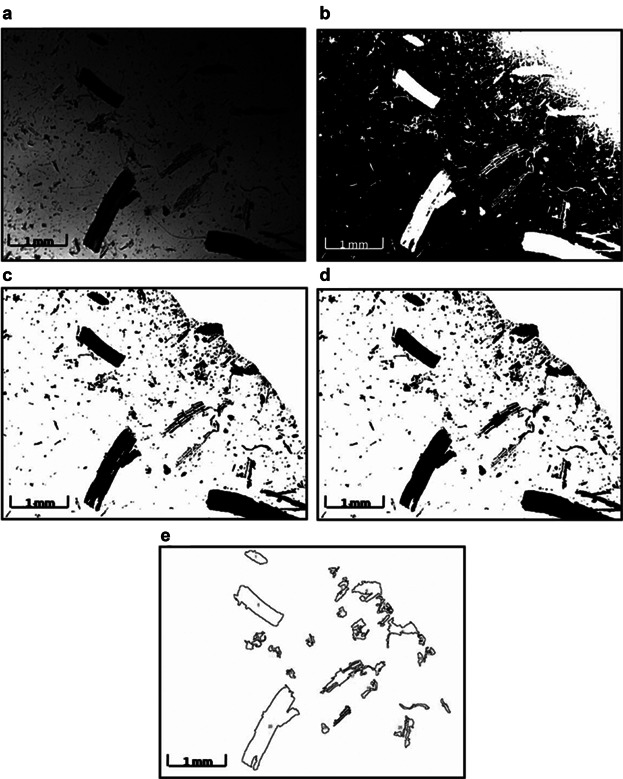
Methodology for root debris size analysis. Light microscopic images were taken at 2.5× magnification for individual samples at each time point. Image J software was used to analyse particle sizes. a–e represents the flow diagram of the image processing algorithm for the analysis. a: A raw image of root debris post-shearing for 240 s, converted to greyscale. b: A thresholded binary image with the particles in white against a black background. c: Using the Freemanual selection tool, the area for analysis has been selected, clearing any shading (here the top right hand corner of the image) caused by shadows of the microscope lens' outer edge, and the image inverted. The particles appear black against a white background. d: Apparent holes or gaps within the root pieces have been filled using the Binary option of the software. e: Using a measured calibration factor of 144 pixels mm^−1^, only root particles of projected area greater than 0.01 mm^2^ were measured, assuming that anything less than this was likely to be adventitious debris and dust, and the feret function (defined by the software as the longest distance between any two points along the selection boundary), considered to be the fragment length.

Approximately 0.35 g of 10 mm roots were sheared in 7 mL of 1× PBS buffer at 75 s^−1^ for 30, 120, 240, or 360 s. At the end of each shearing time, and a control where no shearing was performed (i.e., 0 s), the resulting debris and liquid was centrifuged at 4,000 rpm, for 20 min at 4°C. Shearing experiments were performed in triplicate. The remaining intact roots, that is, those that were still 10 mm in length were blotted dry to remove excess surrounding liquid and weighed. The number of remaining intact roots was calculated as their total mass divided by the average mass of a single intact root (0.00281 ± 0.0002 g). The subsequent estimation of the fraction of remaining intact roots after shear as the final mass of intact roots over the initial root mass (∼0.35 g) was used in Equation 1. To analyze the distribution of the remaining fragments, three images of a 0.1 mL sample of the fragments were imaged using a LEICA DM light microscope (Leica Microsystems Ltd, Heerbrugg, Switzerland) at a magnification of 2.5 times, and root debris lengths characterized using Image J software (Rasband and Bright, 1995). Details of how the software was used to measure fragment length is explained in the legend of Figure 3. This analysis was done in triplicate and the measurements combined to give the fragment length distributions. The frequency of fragments at each length was determined by counting the number of fragments of each length, using the “COUNTIF” function in Excel 2007. Since the sampled images represented a total of 0.3 mL of the total sample in the shearing device, the numbers of each fragment length was multiplied by 20 for an approximate total number in the 6 mL sample (7 mL total volume but 1 mL removed immediately after shearing and centrifugation for IgG analysis).

For IgG analysis, 1 mL of the supernatant was analyzed for IgG1 (Guy's 13 MAb) content using ELISA measurements in duplicate, as described elsewhere (Hassan et al., 2008a). This was repeated in triplicate for each shearing time. As there is variation in IgG1 expression from plant to plant, it was necessary to use grinding in liquid nitrogen as a control from root samples harvested from the same plants. This was done using 0.03 g root pieces in 600 μL of PBS.

### Theory

Due to the macroscopic nature of tobacco roots with lengths of the same order as the impeller size, it seemed likely that breakage mechanisms were primarily dependent on inertial forces, such as collisions of the particles with each other or the impeller. Root–root collisions were also discounted as a major cause of breakage since the suspensions used were dilute. Collisions of roots with the shearing device walls, where the flow is low, were also likely to be less significant. Thus, it was decided *a priori* that the dominant mechanism of damage of roots in the shearing device was collisions with the impeller. There are some examples in the literature where the particles are fragile and undergo break-up by collision with impeller blades and within these solid-liquid mixing systems, the distribution of the impact velocities of the particles is dependent on both their mass and size in addition to local hydrodynamic conditions near the impeller blades (Kee and Rielly, 2004). Here, it was assumed that collision of roots with the impeller caused complete fragmentation of the root into debris. This hypothesis was tested by examining the resulting particle size distributions. It was assumed that all unbroken roots were of length *L*_0_ and there were initially *n*_0_ of these per unit volume, and *n* after shearing for time *t*; a sub-population of roots could not be broken under the prevailing conditions, fraction *u*; breakage of roots on collision with the impeller was assumed to be by complete disintegration into small fragments and there were no fragments initially; passage of the suspension through the impeller had a frequency 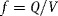 where *Q* was the flow rate of suspension through the impeller and *V* was the volume of the suspension (and chamber); and the combined collision and breakage effectiveness factor was *η*, was a constant. Hence the number of unbroken roots *n* at shearing time *t* is given by:
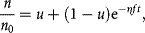
1that is (breakable) root breakage is first order in (breakable) root number. Equation 1 was tested experimentally. The flowrate of the suspension through the impeller was derived from the impeller flow number, Fl:
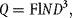
2where *D* is the impeller diameter and *N* its rotational speed. Although the flow pattern in the shearing device is unknown, it was assumed here that it was radial and that: 
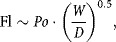
3where *Po* is the impeller Power number (approximating from an expression in Platzer and Noll, 1983), and *W* is the blade thickness. Hence 
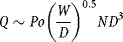
and 
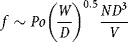
4

Provided the Power number is measured, it is possible to estimate *f* from Equation 4. For the purposes of this work, it was not worthwhile to try to find *f* more precisely as its main use was to estimate and check the reasonableness of the combined collision and breakage effectiveness factor, *η*.

## Nomenclature

*D*: impeller diameter (m)
*f*: suspension passage frequency through the impeller
Fl: impeller Flow number
*n*: number of intact roots
*n*_0_: initial number of intact roots
*N*: impeller speed (s^−1^)
*Po*: impeller Power number
*Q*: flow rate of suspension through he impeller (m^3^ s^−1^)
*t*: shearing time (s)
*u*: fraction of unbreakable roots
*V*: volume of the suspension (m^3^)
*W*: blade thickness (m)
*η*: combined collision and breakage effectiveness factor
